# Ptaquiloside and Pterosin B Levels in Mature Green Fronds and Sprouts of *Pteridium arachnoideum*

**DOI:** 10.3390/toxins12050288

**Published:** 2020-05-01

**Authors:** Debora da Silva Freitas Ribeiro, Kelly Moura Keller, Benito Soto-Blanco

**Affiliations:** 1 Centro Universitário de Mineiros (UNIFIMES), Rua 22-Setor Aeroporto, Mineiros GO 75833-130, Brazil; deboradasfr@unifimes.edu.br; 2 Department of Preventive Veterinary Medicine, Veterinary School, Universidade Federal de Minas Gerais (UFMG), Av. Antônio Carlos 6627, Belo Horizonte MG 30123-970, Brasil; kelly.medvet@gmail.com; 3 Department of Veterinary Clinics and Surgery, Veterinary School, Universidade Federal de Minas Gerais (UFMG), Av. Antônio Carlos 6627, Belo Horizonte MG 30123-970, Brasil

**Keywords:** *Pteridium aquilinum*, bracken fern, poisonous plants, plant toxins

## Abstract

*Pteridium arachnoideum*, a fern of the *Pteridium aquilinum* species complex found in South America, is responsible for several different syndromes of poisoning. Cases of bovine enzootic hematuria and upper alimentary squamous cell carcinoma are both frequent occurrences in Brazil, whereas only bovine enzootic hematuria is noted with any frequency around the world. The reason for the high frequency of upper alimentary squamous cell carcinoma in Brazil is not currently known. One possible explanation may be the higher levels of ptaquiloside and pterosin B in Brazilian *Pteridium* than those present in the plant in other countries. However, these levels have not yet been determined in *P. arachnoideum*. Thus, the present study aimed to measure and compare ptaquiloside and pterosin B levels in mature green fronds and sprouts of *P. arachnoideum* collected from different locations in Brazil. Samples of *P. arachnoideum* were collected from the states of Minas Gerais and Rio Grande do Sul. A total of 28 mature leaf samples and 23 sprout samples were used. The mean concentrations of ptaquiloside and pterosin B present in the mature green fronds of *P. arachnoideum* ranged from 2.49 to 2.75 mg/g and 0.68 to 0.88 mg/g, respectively; in *P. arachnoideum* sprouts, mean concentrations of ptaquiloside and pterosin B ranged from 12.47 to 18.81 mg/g, and 4.03 to 10.42 mg/g for ptaquiloside and pterosin B, respectively. Thus, ptaquiloside and pterosin B levels in *P. arachnoideum* samples collected in Brazil were higher in sprouts than in mature green fronds, as observed in other countries. However, there was no variation in ptaquiloside levels among plants collected from different cities in Brazil. The high frequency of upper alimentary squamous cell carcinoma in Brazilian cattle may not be attributed to greater levels of ptaquiloside and pterosin B in *P. arachnoideum* than in other *Pteridium* species in other countries.

## 1. Introduction

*Pteridium arachnoideum* (Kaulf.) of the Dennstaedtiaceae family is a fern of the *Pteridium aquilinum* species complex found in South America [1,2]. The consumption of this plant can result in human and animal poisoning. In animals, toxic effects vary according to the animal species, the dose ingested, and the time of consumption. The syndromes that can result from *Pteridium* ingestion include cyanide intoxication, thiamine deficiency, progressive retinal degeneration, acute hemorrhagic syndrome or hemorrhagic diathesis, bovine enzootic hematuria, and upper alimentary squamous cell carcinoma. In Brazil, only the last three syndromes have been reported in cattle [3,4,5]. In addition to poisoning the cattle, the consumption of the plant by these animals presents a potential risk for humans, since residues of ptaquiloside can be found in milk [6,7,8] and meat [9,10].

Several chemical compounds have been identified in *Pteridium* ferns, the main one being a norsesquiterpenoid glycoside called ptaquiloside, which has been implicated in cattle poisoning and human cancer [3,4]. Ptaquiloside has proven carcinogenic activity: under alkaline conditions, the ptaquiloside is converted into dienone, a reactive compound capable of forming DNA adducts [11,12]. Viruses, such as papillomavirus, might act as co-carcinogens [13]. In addition, ptaquiloside likely has an immunotoxic effect, as experimental studies with mice reported repression of the cytotoxic activity of natural killer cells [14,15]. However, ptaquiloside levels have not yet been determined in *P. arachnoideum*.

Cases of bovine enzootic hematuria and upper alimentary squamous cell carcinoma are both frequent occurrences in Brazil, whereas only bovine enzootic hematuria is noted with any frequency around the world [3,4,5]. The reason for the high frequency of occurrences of upper alimentary squamous cell carcinoma in Brazil is not known. One possible explanation may be the higher levels of ptaquiloside and pterosin B in Brazilian *Pteridium* than those present in the plant in other countries. However, these levels have not yet been determined in Brazilian *P. arachnoideum*. Thus, the present study aimed to measure and compare the levels of ptaquiloside and pterosin B in mature green fronds and sprouts of *P. arachnoideum* collected from different locations in Brazil.

## 2. Results

Fronds and sprouts from *P. arachnoideum* were collected in two states in South and Southeast Brazil and showed concentrations of ptaquiloside in the ranges of 1.36–3.63 mg/g and 5.19–21.2 mg/g, respectively; these fronds and sprouts showed concentrations of pterosin B in the ranges of 0.42–1.33 mg/g and 2.32–10.7 mg/g, respectively (Table 1). Plant samples were collected in the municipalities of Conselheiro Lafaiete, Esmeraldas, and Ouro Branco in the state of Minas Gerais (MG), Southeast Brazil, and in the municipalities of Canela and Nova Petrópolis in the state of Rio Grande do Sul (RS), South Brazil. There were no significant differences between the levels of ptaquiloside and pterosin B in the mature green fronds collected in different municipalities and states. The levels of ptaquiloside in the sprouts also did not show a significant difference between the five municipalities where the plant was collected. However, the levels of pterosin B differed between the two states and between the municipalities of Esmeraldas (MG) and Canela (RS).

**Figure 1:**
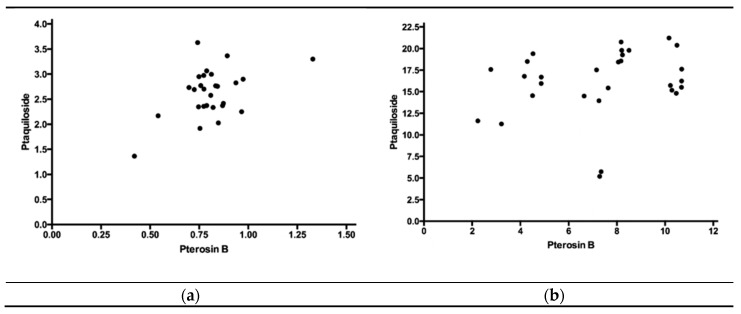
Pearson correlation test between the concentrations of ptaquiloside and pterosin B in *Pteridium arachnoideum*, with significant positive correlation in the mature green fronds (**a**) but no correlation in the sprouts (**b**).

The Pearson correlation test (Figure 1) revealed a significant positive correlation between the concentrations of ptaquiloside and pterosin B in the mature green fronds of *P. arachnoideum* (R = 0.4704, 95% confidence interval: 0.1181 to 0.7176). On the other hand, there was no correlation between the values obtained in the sprouts of this species (R = 0.2213, 95% confidence interval: 0.1580 to 0.5437).

## 3. Discussion

The mean concentrations of ptaquiloside present in the mature green fronds of *P. arachnoideum* ranged from 2.49 to 2.75 mg/g, and those of pterosin B ranged from 0.68 to 0.88 mg/g. These results were in line with those found by Agnew and Lauren [16], where 2.30 mg/g of ptaquiloside and 0.78 mg/g of pterosin B were observed in mature fronds of *P. esculentum* in New Zealand. It was also similar to data from Alonso-Amelot et al. [17] in mature fronds of *P. aquilinum* in Venezuela, with ptaquiloside and pterosin B levels ranging from 1.78 to 1.96 mg/g and 0.54 to 0.79 mg/g, respectively.

Our results also corroborate the findings of Rasmussen et al. [18] with *P. aquilinum* in Denmark, which showed ptaquiloside content in fronds ranging between 0.21 and 2.14 mg/g. In mature fronds in Scotland, the concentration of ptaquiloside ranged from 0.09 to 2.45 mg/g [19]. However, our results presented higher levels than those from mature *P. aquilinum* from the Kullu district in India, in which there was 0.035 ± 0.2 mg/g of ptaquiloside but no detection of pterosin B [20].

By analyzing the samples of *P. arachnoideum* sprouts, we obtained mean values for ptaquiloside of between 12.5 and 18.8 mg/g and pterosin B values that ranged from 4.03 to 10.4 mg/g. These results differ from the study by Alonso-Amelot et al. [17], who found levels of ptaquiloside and pterosin B ranging from 1.88 to 2.34 mg/g and 0.69 to 0.80 mg/g, respectively, in samples from *P. aquilinum* sprouts in Venezuela. Our numbers also differed from Pinto et al. [21], who found ptaquiloside values from 3.79 to 6.53 mg/g in *P. aquilinum* sprouts in São Miguel Island, Azores, Portugal, but did not detect pterosin B. However, our results corroborate with those of Smith et al. [22], who found a range of 0.20 to 12.9 mg/g of ptaquiloside in *Pteridium* in Australia.

Tobar et al. [23] found differences in the concentrations of ptaquiloside in the *P. arachnoideum* collected in three different regions of Ecuador. In fresh young leaves, the value found was 0.59 mg/g, but in the dry young leaves, the value increased to 2.14 mg/g. In fresh mature fronds, the value found was 0.56 mg/g, but in the dry mature fronds, the value increased to 1.827 mg/g. However, these authors found no statistical differences in geographical variables and growth stages. This absence of geographical interference on the concentrations of ptaquiloside was in agreement with both the findings of Rasmussen et al. [24] and those in our study.

The determination of ptaquiloside and pterosin B levels in our study was performed using HPLC with UV detection, first determining pterosin B levels and then converting ptaquiloside into pterosin B. Most of the studies mentioned in the comparisons of our results used the same procedure [16,17,18,19,23,24]. Two of the studies performed direct measurement of both ptaquiloside and pterosin B levels, one of them using HPLC with UV detection [22] and the other one using LC-MS [20]. Thus, the differences in analytical methodology might have a slight impact on results.

The amount of ptaquiloside in the leaves of *Pteridium* spp. is highly variable [19,25]. The highest concentrations of ptaquiloside occur in young sprouts when they emerge from the soil at the beginning of sprouting. Then, with growth, the concentration decreases, with an estimated loss of 10% to 20% of the maximum level [19,26,27]. Mature fronds with very low concentrations of ptaquiloside suggest the loss of toxin during plant aging or even senescence [25]. It has been suggested that, at the end of the plant’s growth, part of the ptaquiloside is transferred to the rhizomes [19]. It has also been put forward that a relevant factor for fern death is the decrease in ptaquiloside concentration in the leaves [28]. Our study showed a significant positive correlation between the concentrations of ptaquiloside and pterosin B in the mature green fronds, but this correlation was not seen in the sprouts. This finding has not been described in earlier literature, and it was not possible to determine its physiological mechanism.

Our results showed that the high frequency of upper alimentary squamous cell carcinoma in Brazilian cattle may not be attributable to the presence of greater levels of ptaquiloside and pterosin B in *P. arachnoideum* when compared to levels found in the plant in other countries. Another hypothesis is the interaction between ptaquiloside consumption and the bovine papillomavirus type four [21]. However, this hypothesis has not yet been tested.

## 4. Conclusions

In the samples of *P. arachnoideum* collected in Brazil, the levels of ptaquiloside and pterosin B were higher in sprouts than in mature green fronds, as observed in other countries. However, there was no variation in ptaquiloside levels between plants collected from different cities in Brazil. The high frequency of upper alimentary squamous cell carcinoma in Brazilian cattle may not be attributed to the existence of greater levels of ptaquiloside and pterosin B in *P. arachnoideum* in Brazil, in comparison to other *Pteridium* species in other countries.

## 5. Materials and Methods

### 5.1. Plant Samples

In total, 28 samples of mature green fronds without spores and 29 samples of sprouts of *P. arachnoideum* were collected from the municipalities of Conselheiro Lafaiete, Esmeraldas, and Ouro Branco in the state of Minas Gerais (MG), Southeast Brazil, and the municipalities of Canela and Nova Petrópolis in the state of Rio Grande do Sul (RS), South Brazil. Both regions suffer from spontaneous outbreaks of *P. arachnoideum* poisoning in cattle.

### 5.2. Obtaining the Pterosin b Analytical Standard

A total of 200 g of fresh sprouts of *P. arachnoideum* was collected in the rural area of the municipality of Santana dos Montes, MG, Brazil. These sprouts were mashed in 1 L of distilled water, and the mixture was kept in the dark at 15 °C for 2 h. The obtained aqueous extract was filtered through a cheesecloth filter, and the plant material was re-extracted with 500 mL of distilled water. Then, the extracts were mixed and 25 g of Diaion resin was added. After 15 min of homogenization in a magnetic stirrer, the solution was filtered through a cloth filter.

The aqueous extract was transferred to a separatory funnel and partitioned three times with 300 mL of dichloromethane each time. The dichloromethane fractions were separated, filtered over sodium sulfate to remove water residues, and combined for further removal of the solvent, which took place in a rotary evaporator, with a maximum temperature of 40 °C. The residue was solubilized with 20 mL of chloroform, then 60 mL of hexane was added, and the mixture was placed in the refrigerator at 2–8 °C for 24 h, forming a supernatant and a sediment. The supernatant was separated and dried at room temperature, with a yield of 99.3 mg.

The residue obtained was analyzed by thin-layer chromatography (TLC) on silica gel 60 plates (Silica-Gel 60 F254, Merck, Darmstadt, Germany), developed in a mobile phase of dichloromethane:acetone (7:3), and then evaluated for fluorescence under UV light (360 nm). Pterosin B was identified by strong fluorescence in the range of Rf 0.8.

The residue obtained and the pterosin B isolated by TLC were evaluated using a high-performance liquid chromatography system coupled to an ultraviolet detector (HPLC-UV). For this system, an ODS column (Shim-pack CLC-ODS [M], Shimadzu, Kyoto, Japan) was used, and the mobile phase consisted of acetonitrile:methanol:water mixed with 1 mM acetic acid (25:25:50) in isocratic mode, at a flow rate of 1 mL/minute, with determination at 230 nm. The peak of pterosin B occurred in approximately 5.0–5.5 min. The purity determined by HPLC of the residue was 71.6%, while the amount that was isolated in the TLC was greater than 90%.

The chemical identity of the obtained pterosin B was confirmed by mass spectrometry, using an Acquity liquid chromatography (UPLC) system (Waters, Milford, MA, USA) coupled to a hybrid mass spectrometer (MS) Xevo G2-S QTof (Waters, Milford, MA, USA) [29]. Chromatographic separation was performed using an Acquity UPLC BEH C18 column (2.1 × 100 mm, particle diameter of 1.7 µm) at 45 °C and sample at 15 °C. The injected volume was 10 µL, and the mobile phase consisted of water with 0.1% formic acid (A) and methanol with 0.1% formic acid (B) at a flow rate of 0.5 mL/min. Elution was carried out in gradient mode according to the following intervals: 0–3.0 min, 75–50% A; 3.0–3.2 min, 50–5% A; 3.2–3.4 min, 5% A; 3.4–3.5 min, 5–75% A; 3.5–4.0 min, 75% A. Mass analysis was obtained by electrospray ionization in positive mode (ESI+), capillary voltage of 3.0 kV, cone voltage of 40.0 V, source offset of 80 V, desolvation gas temperature of 150 °C, and source temperature of 80 °C. The flow of the desolvation gas was 600 L/h of nitrogen. Data acquisition was performed using the MSᴱ method in positive mode, alternating high and low energy in the collision chamber. The settable processing option for confirmation of the pterosin B ion was *m/z* 219.138 [M+H]^+^. MassLynx 4.1 software was used as a control instrument to detect and integrate the peaks.

### 5.3. Detection of Pterosin B and Ptaquiloside in the Samples

All samples were processed and analyzed in duplicate. The method of extracting the samples was adapted from Alonso-Amelot et al. [17]. In a 250 mL Erlenmeyer flask, 1 g of crushed dry plant material and 100 mL of distilled water were added, and the flask was kept in the dark for 2 h under agitation. The obtained aqueous extract was filtered through a paper filter. For the extraction of pterosin B from the samples, 20 mL of the aqueous extract were then transferred to a 50 mL Erlenmeyer flask. Three extractions were made with 10 mL of dichloromethane, and the samples were filtered and dried at room temperature.

To extract ptaquiloside from the samples, 20 mL of the aqueous extract was transferred to another fraction, and 5 mL of 0.04 M potassium hydroxide solution (KOH) was added. This solution was maintained in a water bath at 40 °C for 2 h. Three extractions were made with dichloromethane, and the samples were filtered and dried at room temperature.

For sample injection in HPLC-UV, each sample was reconstituted with 500 mL of methanol. The detection of pterosin B and ptaquiloside in the samples was evaluated by detection with HPLC-UV, according to the technique proposed by Agnew and Lauren [16]. Aliquots of 20 μL were injected and evaluated in an isocratic flow of 1.2 mL/min. For separation, an ODS column (Shim-pack CLC-ODS (M)), with an acetonitrile:methanol:water with 1 mM acetic acid (25:25:50) mobile phase was used. For detection, a fixed 230 nm configuration was used. For calibration, a six-point calibration curve (0.156, 0.31, 0.625, 1.25, 2.5, and 5.0 mmol/L) of the pterosin B standard was used.

The analytical method using HPLC-UV was validated [30], and presented adequate performance criteria for detecting and quantifying pterosin B. The linear regression equation was y = 1610778x + 428867, and the correlation coefficient of the analytical curve was 0.996. The detection and quantification limits for pterosin B were 0.04 and 0.16 mmol/L, respectively.

### 5.4. Statistical Analysis

The results are presented as averages followed by the respective standard deviations. The comparison of ptaquiloside and pterosin B levels between different cities was made using the Kruskal−Wallis test followed by Dunn’s multiple comparison test, while the Mann−Whitney *U* test was used to compare results between states. The determination of the correlation between the levels of ptaquiloside and pterosin B in each plant was carried out using Pearson’s correlation test. The level of statistical significance was set at *p* < 0.05.

## Figures and Tables

**Table 1:** Concentrations (in mg/g) of ptaquiloside and pterosin B in the mature green fronds (without spores) and sprouts of *Pteridium arachnoideum* from different regions of Brazil. Data are presented as mean ± standard deviation (n).

Place of Collection	Mature Green Fronds		Sprouts	
	Ptaquiloside	Pterosin B	Ptaquiloside	Pterosin B
Conselheiro Lafaiete	2.49 ± 0.44 (4)	0.86 ± 0.09 (4)	18.81 ± 2.34 (5)	8.14 ± 0.36 (5) ^a,b^
Esmeraldas	2.75 ± 0.84 (5)	0.68 ± 0.15 (5)	16.38 ± 2.44 (8)	4.03 ± 0.98 (8) ^a^
Ouro Branco	2.66 ± 0.44 (7)	0.88 ± 0.20 (7)	16.51 ± 3.54 (4)	6.86 ± 2.14 (4) ^a,b^
Minas Gerais state ^1^	2.65 ± 0.56 (16)	0.82 ± 0.18 (16)	16.99 ± 2.80 (17)	5.83 ± 2.21 (17) ^A^
Canela	2.51 ± 0.38 (6)	0.82 ± 0.05 (6)	17.37 ± 2.69 (6)	10.42 ± 0.23 (6) ^b^
Nova Petrópolis	2.68 ± 0.27 (6)	0.75 ± 0.13 (6)	12.47 ± 5.62 (6)	8.37 ± 1.71 (6) ^a,b^
Rio Grande do Sul state ^2^	2.61 ± 0.32 (12)	0.78 ± 0.10 (12)	14.92 ± 4.92 (12)	9.40 ± 1.58 (12) ^B^
All regions ^3^	2.63 ± 0.47 (28)	0.80 ± 0.15 (28)	16.14 ± 3.88 (29)	7.30 ± 2.64 (29)

^a,b^ Different letters in the same column indicate significant difference (*p* < 0.05, Kruskal−Wallis test followed by Dunn’s multiple comparisons test); ^A,B^ Different letters in the same column indicate significant difference (*p* < 0.05, Mann−Whitney test). ^1^ Average data from Conselheiro Lafaiete, Esmeraldas, and Ouro Branco. ^2^ Average data from Canela and Nova Petrópolis. ^3^ Average data of all five municipalities.
